# Vitamin K (Menaquinone) from marine *Kocuria* sp. RAM1: optimization, characterization and potential in vitro biological activities

**DOI:** 10.1186/s12934-025-02751-8

**Published:** 2025-06-07

**Authors:** Rasha A. Metwally, Nermeen A. El-Sersy, Amany El Sikaily, Soraya A. Sabry, Hanan A. Ghozlan

**Affiliations:** 1 Marine Microbiology Lab, National Institute of Oceanography and Fisheries, NIOF, Alexandria, Egypt; 2 Marine Pollution Lab, National Institute of Oceanography and Fisheries, NIOF, Alexandria, Egypt; 3 Botany & Microbiology Department, Faculty of Science, Alexandria University, Alexandria, Egypt

**Keywords:** Anti-inflammatory, Anticancer, Antidiabetic, Antimicrobial, Antioxidant, *Kocuria*, Menaquinone, Wound healing

## Abstract

**Background:**

Menaquinone (MK), which is also known as vitamin K2, is a kind of lipoquinone that, unlike humans, is biosynthesized in bacteria through a series of steps as a necessary component of their respiratory chain for electron transport among various components of the bacterial cell membrane. MKs are receiving increasing attention as they play several essential biological roles in humans.

**Results:**

In this study, MK was obtained from *Kocuria* sp. RAM1, characterized using UV absorbance, and validated using nuclear magnetic resonance spectroscopy (NMR) and liquid chromatography-electrospray ionization-quadrupole time-of-flight mass spectrometry (LC-ESI-QTOF-MS). The chemical characterization revealed a total of six MK analogues that were identified and confirmed as MK-1, MK-3, MK-5 (H2), MK-7 (H6), MK-8 (H2), and MK-9. Subsequent to the execution of a significant optimization model, a total KMs of 394.69 µg/ml was obtained, with the MK-1 analog being the dominant one. The antibacterial, anti-inflammatory, antioxidant, anticancer, antidiabetic, and wound-healing activities of MKs were evaluated in vitro. As a result, we discovered that MKs have promising findings on the tested in vitro activities.

**Conclusions:**

Our study was made to evaluate MKs obtained from the Red Sea *Kocuria* sp. RAM1 to emphasize their significant role in different biological applications. Therefore, from a therapeutic and medicinal perspective, the extracted MKs are interesting for additional in vivo studies.

**Supplementary Information:**

The online version contains supplementary material available at 10.1186/s12934-025-02751-8.

## Background

The Danish nutritional biochemist Henrik Dam won the Nobel Prize (1943) for his groundbreaking exploration of vitamin K, which had been the subject of years of study [1]. Dam and colleagues proved that bleeding was prevented in chickens through a vitamin K-containing diet and decided to call it the coagulation vitamin. So, vitamin K was named using the initial letter of “Koagulation” [2].

Vitamin K shows up two distinct forms which are K1 and K2. Vitamin K1, which is widely recognized as phylloquinone, is a critical redox cofactor essential for electron transfer in photosystem I and protein disulfide bond creation. It is formed in plants and some cyanobacteria species [3]. Menaquinone (MK) is another name for vitamin K2, is a kind of lipoquinone that contains isoprenoids and is mostly produced by microorganisms. Eubacteria and archaea contain varying lengths of MK analogues of isoprenyl side chains that are either partly or fully saturated [4]. Therefore, the Mk outline is employed in the phenotypic and taxonomy characterization of bacteria [5] and is a necessary component of their respiratory chain for electron transport among the various components of the bacterial cell membrane [6–8]. In contrast to vitamin K's two natural forms, there is an additional form commonly referred to as menadione or K3, which is artificially produced (non-naturally occurring). Because it does not have the isoprenyl side chain, it is a simple form of 1,4-naphthoquinone and is commonly used in pet and animal feed rather than dietary supplements for humans [9].

The rise in the demand for vitamin K worldwide has sparked a lot of interest in innovative production techniques. Vitamin K can be made as a nutraceutical through microbial fermentation, chemical synthesis, and natural extraction. Among the three methods used in the industrial field of natural vitamin K, microbial fermentation is the most ecologically sound process. Furthermore, it is more cost-effective to produce the final products using inexpensive sources [10]. One of the many benefits of microbial biosynthesis is that it allows the synthetic process to occur in an environmentally safe manner by avoiding the use of strong chemicals, solvents, and heavy metals [11]. MK-7 can be synthesized in substantial numbers by the FDA-approved, food-safe *Bacillus subtilis*, which is sourced from the classic Japanese dish natto and is present in the fermented soybeans of the same designation. [12–15].

**Fig. 1:**
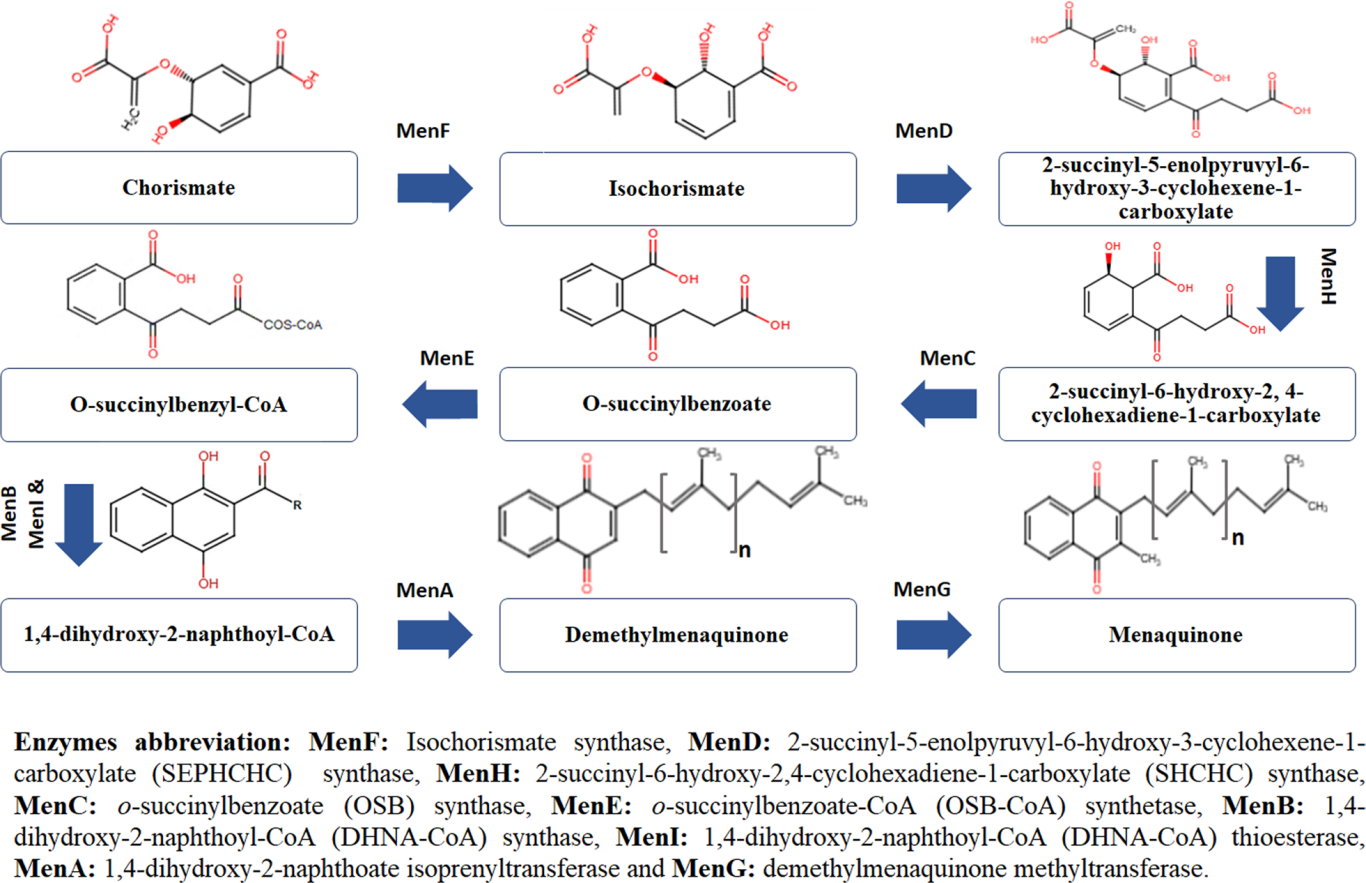
Schematic diagram of MK biosynthesis

In bacteria, it was found that the synthesis of MK involves a series of reactions that initially commence with chorismite, an intermediate generated by the shikimate pathway. Until the last stage, which entails generating MK by appending a methyl group to the naphthalenoid ring's position 3, this process is aided by various enzymes [6, 16] **(**Fig. 1**).**

In humans, unlike bacteria, MK biosynthesis is absent and the respiratory chain's electron carrier is ubiquinone [6, 17]**.** Given the necessity of MK for blood coagulation, bone metabolic functions, cell cycle regulation, blood pressure and coronary heart disease risk reduction [18–21], humans receive it through meals, synthesized by gut microbes in the lower intestine [22, 23]. It has been demonstrated that mammals can use side chain removal and addition to create MK-4 from dietary phylloquinone [24]. Endogenous synthesis provides between 10 and 50% of the MK needed by humans. The MKs generated by the gut microbiota seem to be useful to the host as well as to some microbiomes that need it or its derivatives to grow [25]. Moreover, vitamin K has also been proposed as a potential way to lower the morbidity and death rates attributed to coronavirus disease 2019 (COVID-19) [26–28].

Red sea cucumbers were used to isolate *Kocuria* sp. RAM1 in a previous study. Considering that animal-associated bacterial populations frequently contrast with those found in the water column, exhibiting unique characteristics and synthesizing biologically active compounds of functional and structural significance. *Kocuria* sp. RAM1 exhibited the synthesis of a novel mixture of carotenoid pigments, including 3,4,3ʹ,4ʹ-tetrahydrospirilloxanthin (C42-carotenoids), alongside two C_50_-carotenoids of bisanhydrobacterioruberin and trisanhydrobacterioruberin. This led us to look into other MK extracts from it [29]. Thus, the current work is intended to uncover new marine bacteria as a source of MK production while also screening for possible biological uses.

## Methods

### Strain, cultivation conditions, and MK extraction and purification

From Marsa Alam, Red Sea, Egypt, sea slug *Bohadschia graeffei* samples were collected, from which the bacterium *Kocuria* sp. RAM1 was isolated among other bacteria. Only *Kocuria* sp. RAM1 was determined to produce MK in the cells. Following sterilization, a 1% inoculum is added to a medium containing peptone (5 g/l), yeast extract (2 g/l), beef extract (3 g/l), NaCl (5 g/l), glucose (1 g/l), and MgSO₄ (1 g/l) at 30 °C, 120 rpm, and pH = 7 for 48 h. After centrifuging 100 ml of a 48-h culture at 6000 rpm for 15 min, the cells got cleaned with distilled water. For extraction, the mixture of cells and methanol (100 ml) was mixed vigorously and incubated for 45 min in a 40 °C water bath until complete color extraction. MK is obtained by evaporation of methanol at 40 °C to dryness [29–31]. For purification, the extract was mixed with methanol and combined with petroleum ether in a separating funnel until the two-phase separation. To get rid of any remaining carotenoids, the methanolic yellow phase was collected and repeatedly cleaned with petroleum ether, followed by MK concentration in the methanolic layer at 35 °C.

### Identification of MK

#### UV spectroscopy

The UV–Vis absorbance spectrum was measured by scanning the yellow methanolic fraction between 200 and 600 nm [32].

### Proton nuclear magnetic resonance

Proton nuclear magnetic resonance (^1^H-NMR) was obtained using deuterated methanol (CD₃OD) at 500 MHz (JEOL ECZ) [33].

### LC-ESI-QTOF-MS

The yellow methanolic fraction composition was determined via LC-QTOF-MS (SCIEX X500R QTOF, USA), set by electrospray ionization (ESI) in a positive ionization mode. A 3:1 (v/v) methanol/isopropanol ratio at 0.45 ml/min and a flow at the rate of 8.0 l/min of dry nitrogen gas were both components of the mobile phase. A capillary voltage and a cone voltage were established at 4.5 and 30 V, respectively. The temperature was set at 147 °C, a collision energy of 27 eV, and a range scan of 100–1500 m/z were all used for the mass spectrometry conduction [34]. For quantification of each MK analog, the peaks were integrated, and the area of each peak was used for calculation of MK concentrations in the mixture.

### Statistical optimization of MK

Primarily, the most significant variables impacting MKs using Design Expert V.11 were identified by Plackett–Burman experimental design (PBD) (Table 1) [35]. The model equation was as follows [36]:

1 $${\text{Y}} = \beta_{0} + \sum\nolimits_{i = 1}^{K} {\beta_{i } Xi \left( {i = 1, \ldots ., K} \right)}$$

**Table 1:** PBD high and low variables used to screen for MK production

Independent variables	Code	Experimental value	
		−	+
Peptone (g/l)	A	1.0	9.0
Yeast extract (g/l)	B	1.0	3.0
Beef extract (g/l)	C	1.0	5.0
NaCl (g/l)	D	0.0	10.0
Glucose (g/l)	E	0.5	1.5
MgSO_4_ (g/l)	F	0.5	1.5
pH	G	4.0	10.0
Temperature (°C)	H	30.0	40.0
Agitation (rpm)	J	90.0	150.0
Inoculum size (%)	K	0.5	1.5
Incubation hours (h)	L	24.0	72.0

The model intercept is denoted by β_*0*_. The linear regression coefficient is expressed as β_*i*_, the independent variable level as X_*i*_, the number of variables as K, and the MKs response (µg/ml) as Y.

Following that, four important factors were determined for the observation of MK production: peptone, temperature, agitation, and inoculum size. The use of Design Expert **(**Table 2**)** for central composite inscribed design (CCI) was implemented [37]. The model calculation was conducted using the following equation [38]:

2 $${\text{Y}} = X_{0} + \sum\nolimits_{i = 1}^{K} {\beta_{i} X_{i} } + \sum\nolimits_{i = 1}^{K} {\beta_{ii} X_{i}^{2} } + \sum {\sum\nolimits_{i < j = 1}^{K} {\beta_{ij} X_{i} X_{j} + {\text{E}}} }$$

where Y is MK (µg/ml), the intercept is expressed as X_*0*_; the equation coefficients (linear, quadratic, and interaction) as β_*i*_, β_*ii*_, and β_*ij*_, respectively. Rarameters denoted by coded values are represented as X*i* and X*j*, E signifies the experimental error, and K indicates the parameter number.

**Table 2:** Response surface method (RSM) ranges for the most critical independent variables

Name	Code	Variable levels				
		-α	-1	0	+ 1	+ α
Peptone (g/l)	A	5	6.25	7.5	8.75	10
Temperature (°C)	B	30	31.75	33.5	35.25	37
Agitation (rpm)	C	120	140.00	160.0	180.00	200
Inoculum size (%)	D	1	1.50	2.0	2.50	3

α refers to the distance of each axial point from the center in a central composite design

### Applications of MKs

#### Antibacterial activity

**Fig. 2:**
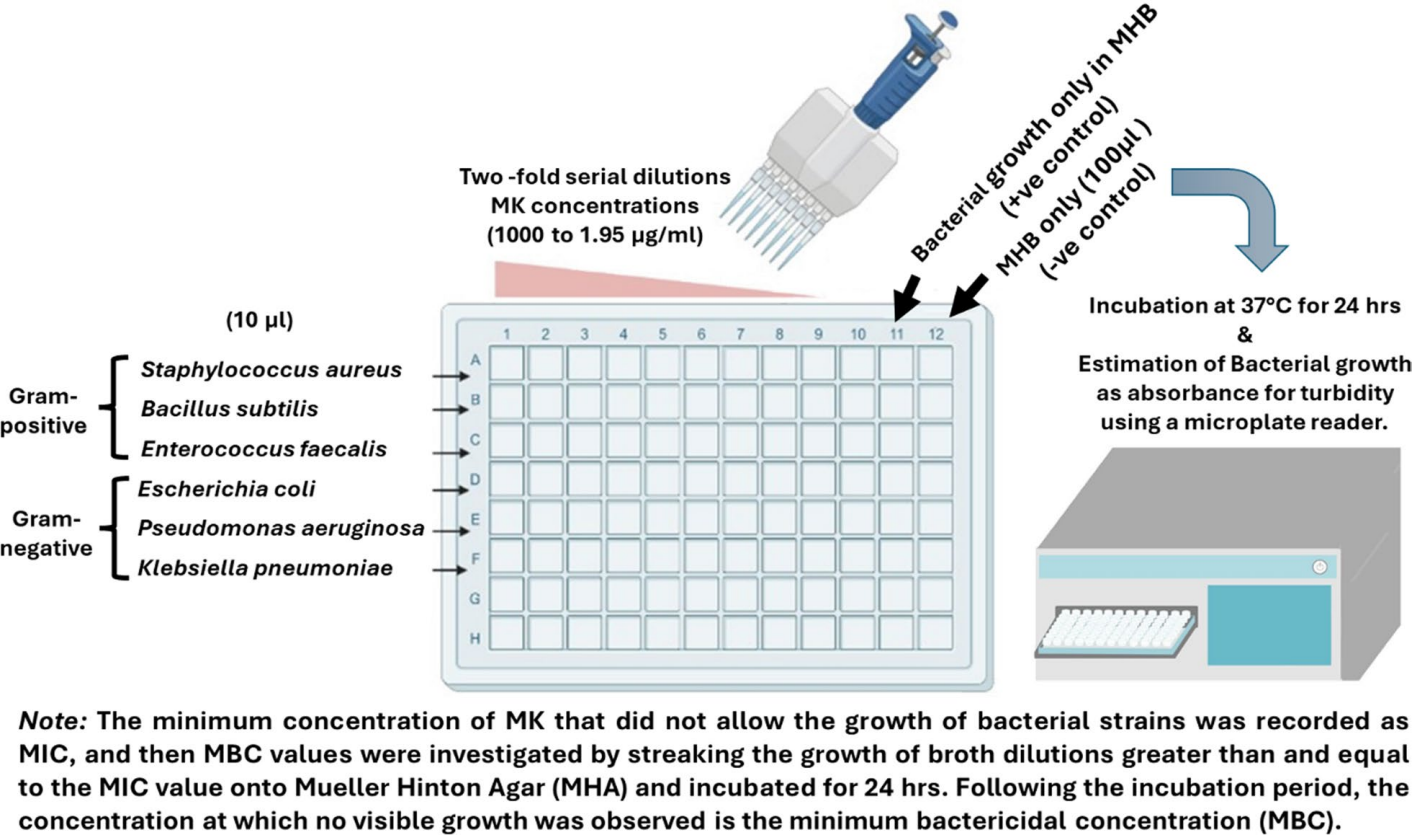
Schematic diagram for MIC and MBC of MK

Three bacterial strains of Gram-positive, in addition to three strains of Gram-negative ones, were chosen for assessing the MK's minimum inhibitory concentration (MIC) and minimum bactericidal concentration (MBC) with the inhibition zone observation [39, 40] **(**Fig. 2**)**.

### Anti-inflammatory potential

MK anti-inflammatory potentials of 100–1000 µg/ml were explored via hypotonicity-induced hemolysis with the use of human red blood cells (RBCs) according to Shinde et al. [41]. RBC membrane rupture was studied in hypotonic and isotonic solutions. In EDTA tubes, RBC samples were collected. Five milliliters of the hypotonic solution containing the test concentration of MK were combined with 30 µl of erythrocyte suspension. The mixtures were centrifuged for five minutes after being incubated for ten minutes at room temperature. The negative and positive controls were distilled water and phosphate buffer saline (PBS), respectively. Hemolysis inhibition % was calculated using the absorbance (Ab) of supernatants at 540 nm.

3 $${\text{MK}}\,{\text{hemolysis inhibition (\%) }} = \left[ {1 - \left( {\frac{{{\text{Ab}}_{2} - {\text{Ab}}_{1} }}{{{\text{Ab}}_{3} - {\text{Ab}}_{1} }}} \right)} \right]*100$$

where the MK and RBC mixture in both PBS and distilled water absorbances are denoted by the letters Ab_1_ and Ab_2_, respectively. Ab_3_ is the absorbance of RBCs in distilled water as control.

### Antioxidant activity

The color intensity at 540 nm of DPPH (1,1-diphenyl-2-picryl hydrazyl) (100 µl, 0.1 mM) was measured after 30 min at the ambient temperature under dark conditions. 100 µl of serial concentrations of Mk (1.95–1000 µg/ml) were tested [42]. The below equation was used for assessing the DPPH-scavenging activity:

4 $${\text{MK DPPH Scavenging activity }}\left( \% \right) = \left( {\frac{{{\text{Ab}}_{{\left( {{\text{Control}}} \right)}} - {\text{Ab}}_{{\left( {{\text{MK}}} \right)}} }}{{{\text{Ab}}_{{\left( {{\text{Control}}} \right)}} }}} \right)*100$$

### Wound healing assay

In Vero cells, the maximum non-toxic dose (MNTD) was estimated by successive doses of MK (31.25–1000 µg/ml) using the MTT (3-[4, 5-dimethylthiazol-2-yl]-2,5-diphenyltetrazolium bromide) assay, as described by Rai et al*.* [43]. The resulting MNTD (125 µg/ml) was used for wound healing using the scratch assay of two types of cells: HSF (human skin fibroblasts) and OEC (oral epithelial cells) [44]. HSF and OEC cells (3 × 10^5^/well) were cultured in DMEM (Dulbecco’s Modified Eagle Medium) with 5% FBS (foetal bovine serum) for twelve hours (37 °C, 5% CO_2_), scratched horizontally, introducing a gap, or wound. Fresh media wells served as a negative control, while test wells contained media and MK’s MNTD [45]. Results were documented as images captured immediately after scratching every 24 h and analyzed by ImageView software v 3.7. The efficiency of MK is calculated as a wound closure% by the following equation:

5 $${\text{MK wound closure }}\left( \% \right) = \frac{{{\text{A}}_{h = 0} - {\text{A}}_{\Delta h} }}{{{\text{A}}_{h = 0} }}*100$$

where A_*h*=*0*_ and A_*∆h*_ refers to the original and the time-changed wound widths measured after hours.

### Anticancer activity

The cytotoxicity of MK against carcinoma of three cell lines was investigated: human breast cancer (MCF-7), human colon carcinoma (Caco-2), and cervical carcinoma (HeLa). A triplicate MTT assay conduction was employed in the calculation of both the IC_50_ and the selectivity index (SI) [46].

### Antidiabetic activity

To assess the antidiabetic efficacy of MKs, different concentrations of MK were evaluated through in vitro α-glucosidase inhibition [47]. A mixture formed by 50 µl of phosphate buffer (100 mM, pH = 6.8), 10 µl of α-glucosidase (1 U/ml), together with 20 µl of different MK concentrations (1000 to 7.81 µg/ml) was incubated for 15 min at 37 °C in a 96-well plate. After that, 20 µl of P-NPG (p-Nitrophenyl beta-D-glucopyranoside, 5 mM) was incorporated as a substrate, and the mixture was incubated for a further 20 min at 37 °C. Ultimately, it is crucial to terminate the reaction by introducing 50 µl of Na₂CO₃ (0.1 M). The absorbance readings at 405 nm, standing for α-glycosidase inhibitory, were recorded with Acarbose as a reference as follows

6 $${\text{MK inhibitory activity }}\left( {\%} \right) = \left( {\frac{{{\text{Ab}}_{Acarbose} - {\text{Ab}}_{MK} }}{{{\text{Ab}}_{Acarbose} }}} \right)*100$$

where both Ab_*Acarbose*_ and Ab_*MK*_ stand for the absorbance of Acarbose and MK, respectively.

### Statistical analysis

Tukey's test and ANOVA were performed at *p* = 0.05 by implementing triplicate experimental data displayed as mean ± SD using SPSS software V 22.0.

## Results

### MK extraction, purification and identification

**Fig. 3:**
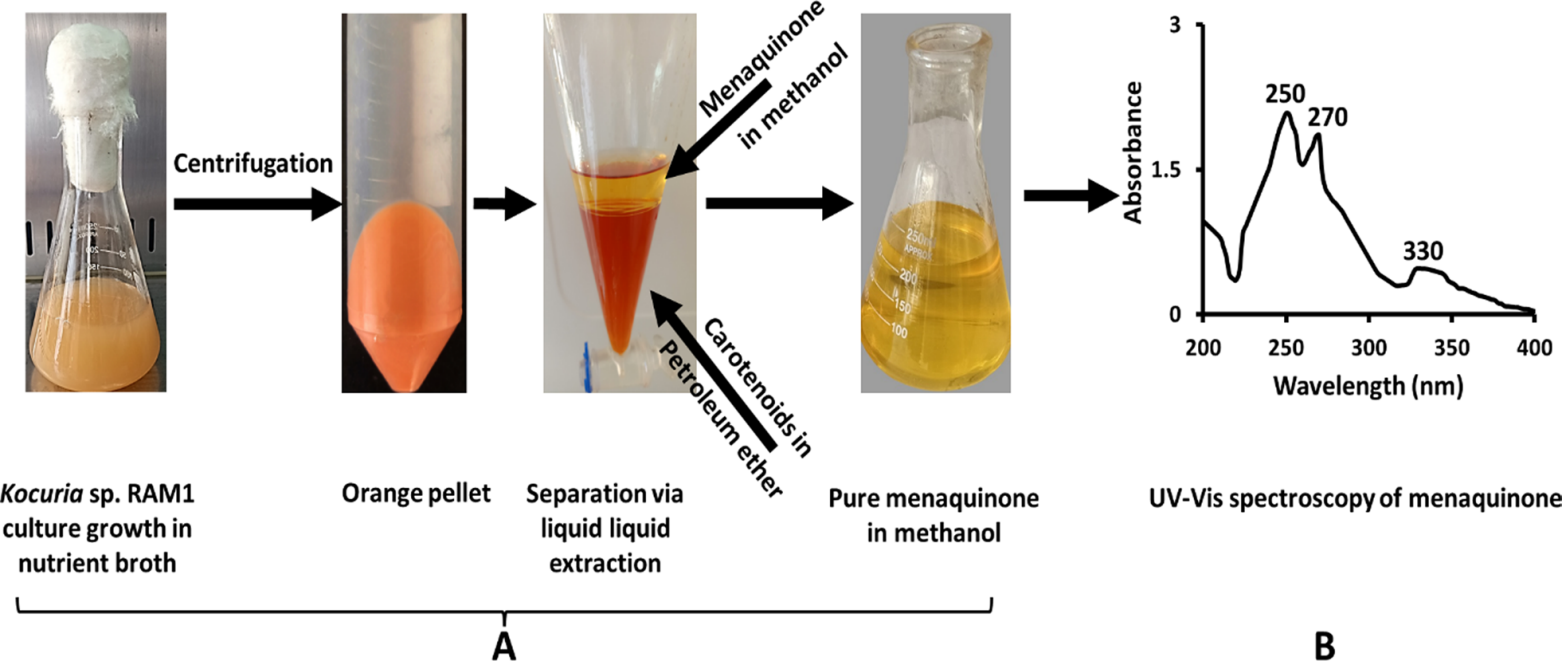
*Kocuria* sp. RAM1 MK extraction and purification (**A**). UV–Vis spectroscopy (**B**)

**Fig. 4:**
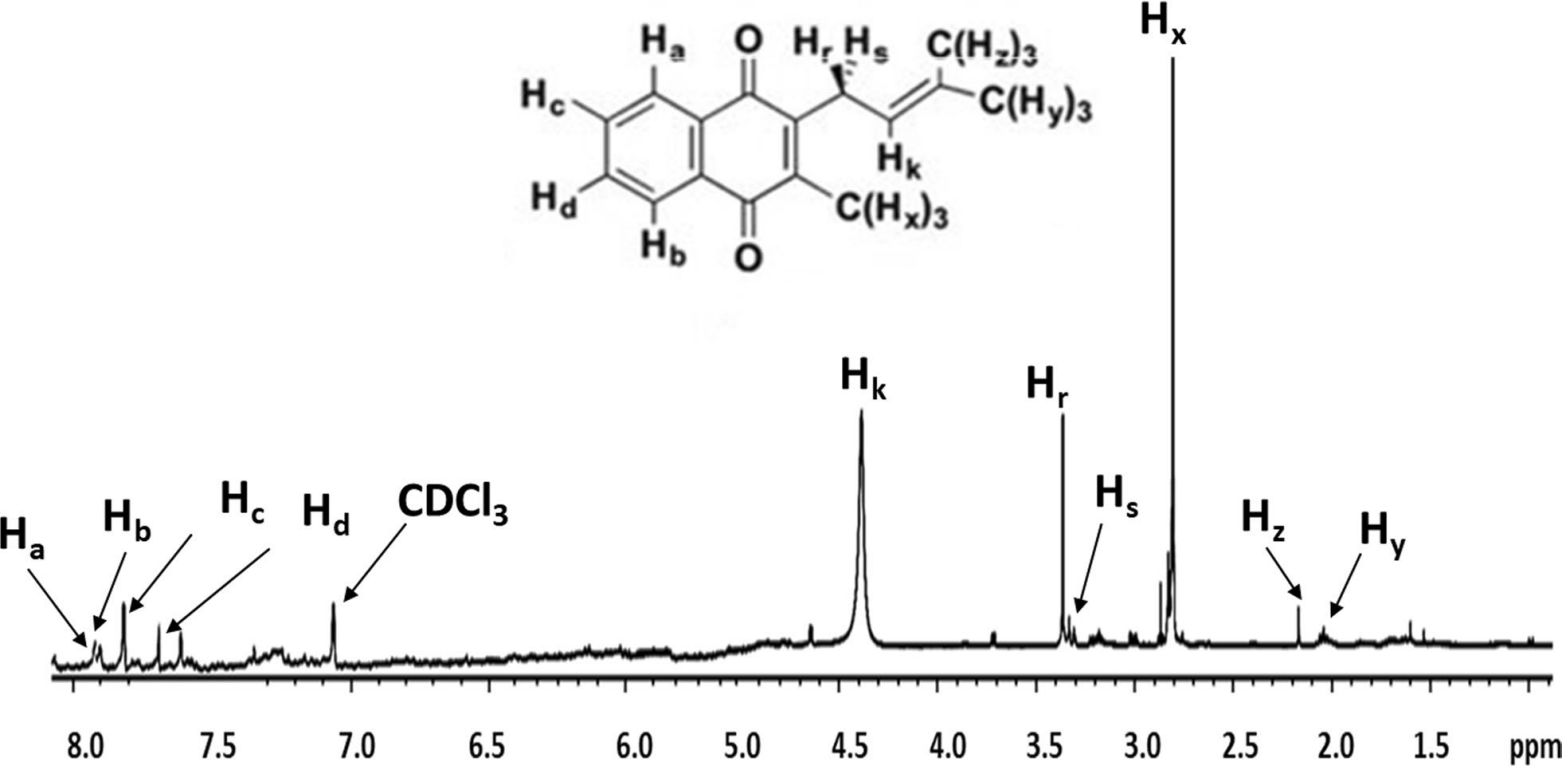
^1−^HNMR analysis of *Kocuria* sp. RAM1 MK

**Fig. 5:**
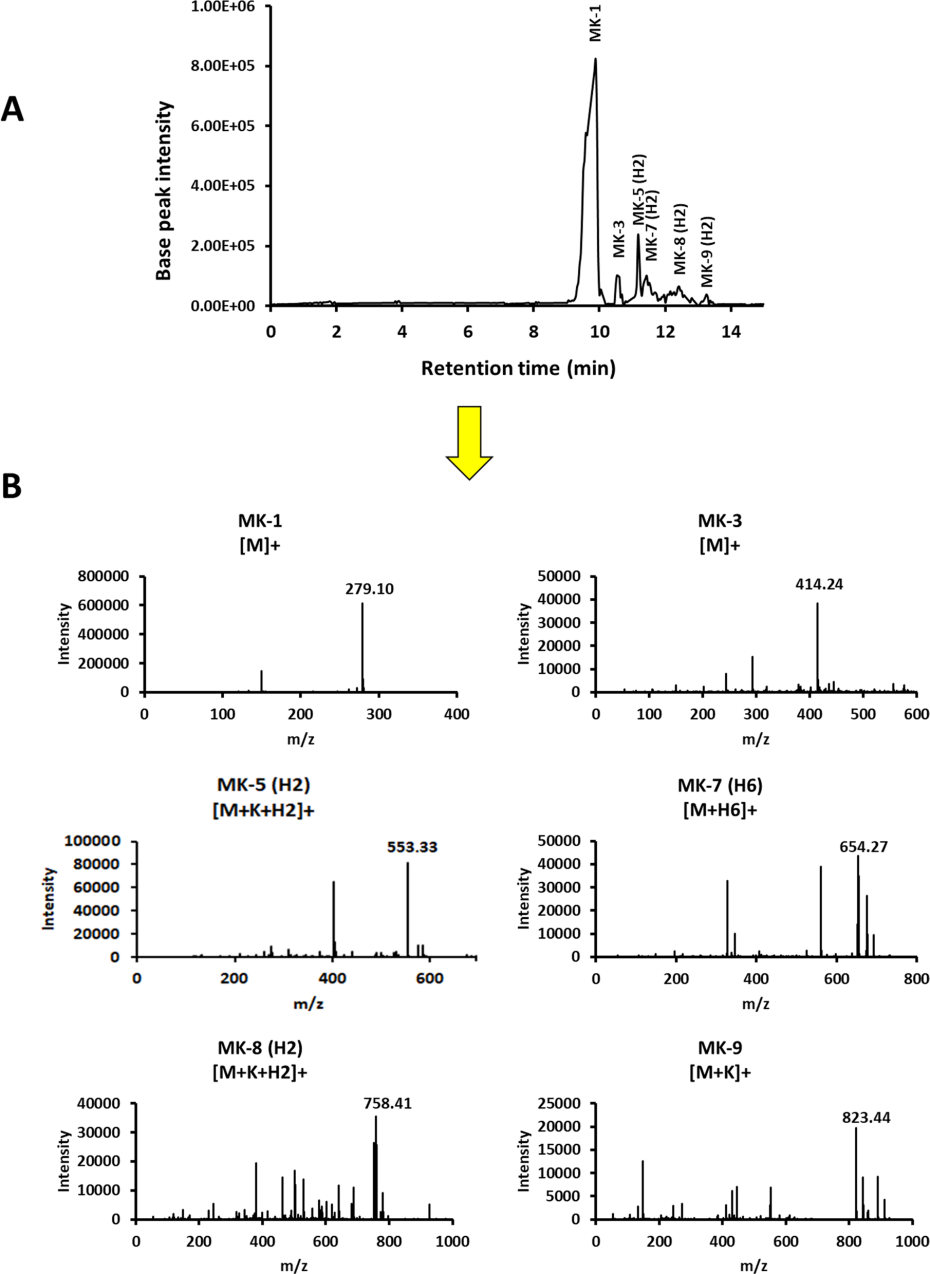
LC-QTOF-MS analysis of *Kocuria* sp. RAM1 of MK (**A**). Mass of MK derivatives (**B**)

**Fig. 6:**
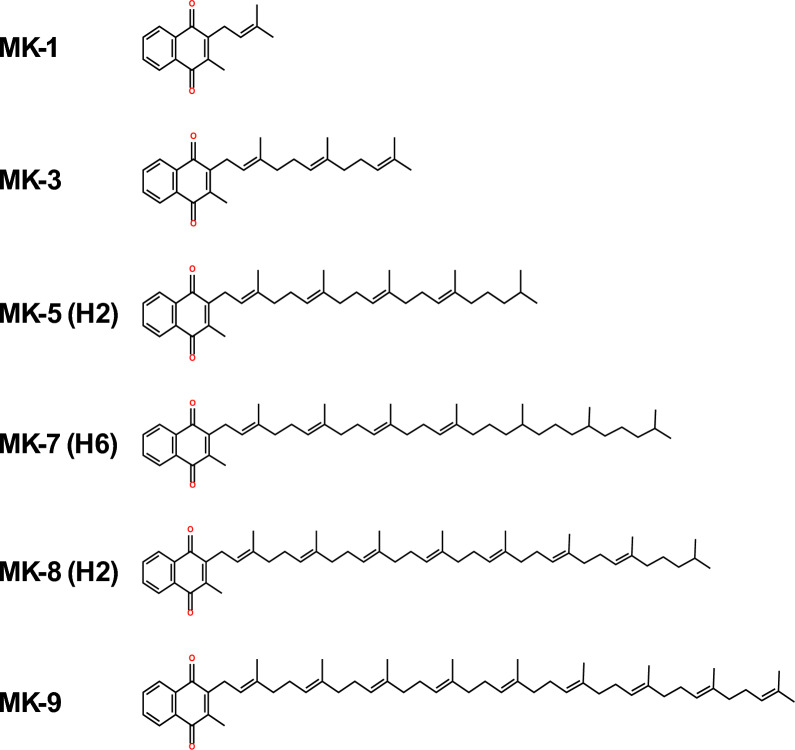
The chemical structures of MK analogs produced by *Kocuria* sp. RAM1

The *Kocuria* sp. RAM1 extract was separated by liquid–liquid extractions into two phases of red and yellow colors (Fig. 3A). In the current study, after drying the yellow methanolic fraction, an oily yellow extract was obtained. The yellow extract’s measured UV–Vis absorbance illustrated distinct peaks at 250, 270 and 330 nm (Fig. 3B). The ^1^H NMR spectroscopic analysis revealed resonances in the 7–8 ppm range confirming a 2-methyl-1,4-naphthoquinione ring identity, with a resonance at 2 ppm of its methyl group protons. In addition, the protons of conjugated chain are represented in the region of 4.8–5.2 ppm, besides the region of 2.1–2.5 ppm of its methyl groups (Fig. 4). The yellow extract’s composition was determined using an LC-ESI-QTOF-MS (Fig. 5A). Six peaks were obtained at retention times (RTs) of 9.7, 10.68, 11.36,11.66, 12.57 and 13.17 min, with molecular ions at m/z 279.10 ([M]^+^), 414.24 ([M]^+^), 553.33 ([M + K + H2]^+^), 654.27 ([M + H6]^+^), 758.41 ([M + K + H2]^+^) and 823.44 ([M + K]^+^), respectively **(**Fig. 5B**).** Referring to the previous details, we could confirm each of the MKs as MK-1 (C_16_H_12_O_2_, 240.115 Da), MK-3 (C_26_H_32_O_2_, 376.24 Da), MK-5 (H2) (C_36_H_50_O_2_, 514.36 Da), MK-7 (H6) (C_46_H_71_O_2_, 654.49 Da), MK-8 (H2) (C_51_H_74_O_2_, 718.55 Da) and MK-9 (C_56_H_80_O_2_, 784.61 Da) (Fig. 6).

### Optimization of MK production and quantification

The eleven variables that may contribute to MK production by *Kocuria* sp. RAM1 have been explored using PBD throughout 12 runs (Table 3). The model explained 91.61% of experimental data variability, with a coefficient of determination of 0.9161, and was correlated with the predicted R^2^ of 0.75 (Table S1). So, the model was statistically significant. Factors like peptone, temperature, agitation, and inoculum size have been suggested for further optimization ( REF _Ref168782750 \h \* MERGEFORMAT Fig. S1). Hence, the model equation for MK production is as follows:

7 $${\text{MK }}\left( {\mu {\text{g}}/{\text{ml}}} \right) \, = {104.6} + {28.80}{\text{ Peptone }} - { 74.14}{\text{ Temperature }} + { 44.69}{\text{ Agitation }} + { 43.51}{\text{ Inoculum size}}$$

**Table 3:** Identification of the key independent variables influencing MK output using the Plackett–Burman design

Run	Independent Variables											
	A	B	C	D	E	F	G	H	J	K	L	MK (μg/ml)
1	1	1	1	−1	−1	−1	−1	−1	1	1	1	283.46
2	−1	1	1	−1	−1	1	1	1	1	−1	−1	20.77
3	−1	1	1	1	1	1	−1	−1	−1	−1	1	61.60
4	1	−1	1	1	1	−1	−1	1	1	−1	−1	7.57
5	1	−1	−1	−1	1	1	1	−1	1	−1	1	240.40
6	−1	−1	−1	1	−1	1	−1	1	1	1	1	60.88
7	1	1	−1	−1	1	1	−1	1	−1	1	−1	64.06
8	1	1	−1	1	−1	−1	1	1	−1	−1	1	15.17
9	−1	−1	−1	−1	−1	−1	−1	−1	−1	−1	−1	17.80
10	−1	1	−1	1	1	−1	1	−1	1	1	−1	279.43
11	1	−1	1	1	−1	1	1	−1	−1	1	−1	186.50
12	−1	−1	1	−1	1	−1	1	1	−1	1	1	11.10

A, B, C, D, E and F (g/l) stand for peptone,** y**east extract, beef extract, NaCl, glucose and MgSO_4_, respectively. G, H, J, K and L stand for pH, temperature (°C), agitation (rpm), inoculum size (%) and incubation period (h), respectively

Thirty runs of RSM were conducted **(**Table 4**)**, showing significant results (R^2^ = 0.9360) (Table S2). Consequently, ANOVA findings were appropriate for expressing the true correlations between MK output and significant factors. The final experimental estimate of MKs is as follows:

8 $${\text{MK}}\,\,{(}\mu {\text{g/ml)}}\,{ = }\, + 189.04\, + \,29.87{\text{A}}\,{ - }\,{74}{\text{.47B}}\,{ + }\,{26}{\text{.24C}}\,{ + }\,{0}{\text{.2308D}}\,{ - }\,{16}{\text{.69AB}}\,{ + 8}{\text{.51AC}}\,{ + }\,{23}{\text{.21AD}}\,{ - }\,{2}{\text{.46BC}}\,{ - }\,{6}{\text{.66BD}}\,{ + 13}{\text{.12CD}}\, - 8.60{\text{A}}^{{2}} \, + 1.38{\text{B}}^{{2}} { + 7}{\text{.12C}}^{{2}} { + }\,{4}{\text{.27D}}^{{2}}$$

where peptone, temperature, agitation, and inoculum size, respectively, are represented by A, B, C, and D.

**Table 4:** RSM of the four important independent variables affecting MK production

Run	Variables		MK (µg/ml)			
	A Peptone (g/l)	B Temperature (°C)	C Agitation (rpm)	D Inoculum Size (%)	Observed Value	Predicted Value
1	0	0	2	0	285.16	270.02
2	0	0	0	0	189.97	189.04
3	−1	−1	−1	−1	233.45	230.36
4	−1	1	−1	−1	123.00	133.06
5	−2	0	0	0	107.91	94.91
6	1	1	−1	−1	102.77	95.98
7	1	−1	−1	−1	245.06	260.05
8	1	1	1	1	158.94	194.10
9	0	0	0	0	186.13	189.04
10	−1	−1	1	1	199.27	238.14
11	−1	1	1	1	135.64	104.32
12	0	0	0	0	180.54	189.04
13	0	0	0	0	222.24	189.04
14	1	−1	1	1	421.07	394.69
15	0	2	0	0	39.34	45.63
16	1	1	−1	1	117.83	103.29
17	−1	−1	−1	1	170.45	171.49
18	1	1	1	−1	151.67	134.31
19	0	0	0	0	222.36	189.04
20	0	0	0	−2	220.20	205.65
21	1	−1	−1	1	284.30	294.01
22	−1	1	−1	1	41.93	47.53
23	0	0	0	2	207.77	206.57
24	1	−1	1	−1	281.77	308.24
25	0	0	0	0	133.00	189.04
26	−1	−1	1	−1	246.31	244.53
27	0	0	−2	0	165.66	165.04
28	2	0	0	0	217.13	214.38
29	0	−2	0	0	365.57	343.53
30	−1	1	1	−1	115.00	137.36

Additionally, in 3D plots, Fig. S2. depicts the two-factor influence of the four significant factors on MK production. Enhanced peptone with lower temperatures boosted MK production. Higher temperatures lead to less production (94.93 µg/ml) (Fig. S2A), while higher agitation speed (180 rpm) and inoculum size percentage resulted in higher MK (251.014 and 237.64 µg/ml, respectively) (Fig. S2B, F & C). The synthesis of MKs was found to be negatively impacted by high temperatures in general (Fig. S2D, E). The desirability function was utilized for optimizing MK, resulting in an optimal output of 394.69 µg/ml with 0.981 as a desirability, despite a 3.693 percentage error (optimal values: peptone = 8.75 g/l, at 180 rpm with inoculum size = 2.5%, at 31.7 °C). Thus, the optimized condition that showed the highest MK production (394.69 µg/ml) was used for characterization and biological applications. Peptone (8.75 g/l), yeast extract (2 g/l), beef extract (3 g/l), NaCl (5 g/l), glucose (5 g/l), and MgSO_4_ (1 g/l) are the ingredients. The mixture is adjusted to pH 7 at 31 °C and 180 rpm, and an inoculum size of 2.50% is used for a 48-h incubation period. The area under each peak of the optimized sample was calculated from the LC chromatogram to determine each analog concentration in the MK in the mixture. It was found that MK-1 makes up about 80.07% (316.03 µg/ml) of the sample, while MK-5 (H2) and MK-7 (H6) have almost the same concentration at 5.57% (21.98 µg/ml) and 5.55% (21.92 µg/ml), respectively. The other analogues of MK-8 (H2), MK-3 and MK-9 represent a lower concentration of 4.02% (15.87 µg/ml), 3.85% (15.20 µg/ml) and 0.93% (3.69 µg/ml), respectively (Table 5).

**Table 5:** LC-QTOF-MS data of the six MKs of *Kocuria* sp. RAM1

Mk	Molecular formula	MWt (Da)	RT	Area (%)	µg/ml
MK-1	C_16_H_12_O_2_	240.115	9.88217	80.07	316.03
MK-3	C_26_H_32_O_2_	376.24	10.5255	3.85	15.20
MK-5 (H2)	C_36_H_50_O_2_	514.36	11.1689	5.57	21.98
MK-7 (H6)	C_46_H_71_O_2_	654.49	11.4339	5.55	21.92
MK-8 (H2)	C_51_H_74_O_2_	718.55	12.4179	4.02	15.87
MK-9	C_56_H_80_O_2_	784.61	13.2505	0.93	3.69
Total				100.00	394.69

### Applications of MKs

#### Antibacterial potential

The six strains’ MIC was found to be 250 to 500 µg/ml, which matches the zone of inhibition findings **(**Table 6**)**. For all tested strains except for *Bacillus subtilis*, the observed MBC value was 1000 µg/ml. The not determined result (ND) of the MBC effect indicates that total lethality requires concentrations exceeding 1000 µg/ml.

**Table 6:** MIC, MBC and zone of inhibition of MK. (ND = Not determined)

	Pathogen	Inhibition Zone (mm)	MK concentration (µg/ ml)	
			MIC	MBC
Gram-positive	*Staphylococcus aureus* (ATCC 25923)	8 ± 0.57	250	1000
	*Bacillus subtilis* (ATCC 6633)	7 ± 1	500	ND
	*Enterococcus faecalis* (ATCC 29212)	12 ± 1	500	1000
Gram-negative	*Escherichia coli* (ATCC 8739)	9 ± 1	250	1000
	*Pseudomonas aeruginosa* (ATCC 9027)	10	250	1000
	*Klebsiella pneumoniae* (ATCC 13883)	12 ± 1.73	500	1000

### Anti-inflammatory potential

The RBC membrane stabilization as an anti-inflammatory potential was increased with a higher MK concentration, providing efficient protection from hypotonicity. Between 100 and 1000 µg/ml, the hemolysis inhibition of the MK varied between 31.70% ± 0.25 and 87.93% ± 0.24, and a concentration of 164.4 ± 0.35 µg/ml inhibited RBCs from rupturing by 50% (IC_50_) (Fig. S3).

### Antioxidant potential

By assessing the antioxidant activity of MKs, the findings showed that as concentrations rose, so did their capacity to scavenge DPPH. The antioxidant activity ranged from 21.37% ± 0.45 (1.95 µg/ml) to 83.56% ± 0.14 (1000 µg/ml). 37.35 µg/ml of MK was determined to lower the DPPH concentration by 50% (IC_50_) (Fig. S4).

### The healing of wounds efficiency

**Fig. 7:**
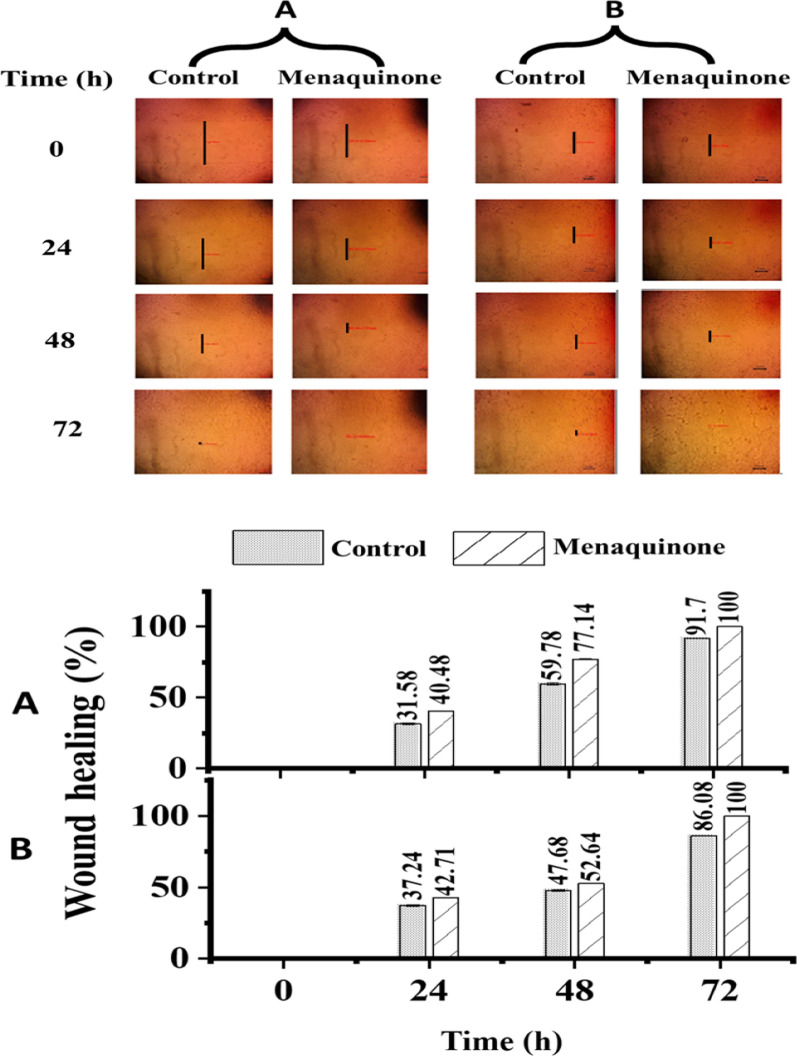
Effect of MK on wound closure on HSF (**A**) and OEC (**B**) cells every 24 h. Mean ± SD _(n=3)_ is represented in the data

In our *in vitro* study, Vero cells were selected because of their availability, ease of handling, and toxicity sensitivity. At both 500 and 1000 µg/ml, a noticeable reduction in cell viability was observed (Fig. S5A). MK IC_50_ was found to be 430.8 µg/ml (Fig. S5B), while MNTD 125 µg/ml was estimated in its presence. The treatment of wounds in HSF, in addition to OEC, was tested at 125 µg/ml (MNTD) of MK. Wound size shrinkage was recorded every 24 h (Fig. 7). In comparison with the control, MK boosted the healing of both HSF and OEC cells. In the case of HSF cells (Fig. 7A), the initial data collected after 24 h revealed that wound closure was 40.48% greater than that of the control (31.58%). The reduction continued after 48 h by 77.14% and completely closed after 72 h, whereas the wounds of the control samples closed entirely after 96 h. Similarly, OEC showed an improved wound closure rate when treated with MK in comparison with the control samples (Fig. 7B). MK enhanced the wound closure rates after 48 h, recording 52.64%, compared to the control (47.68%), with full healing observed after 72 h, earlier than the 96-h control wound closure. These results demonstrate the possibility of applying MK in the wound-healing process.

### Anticancer potential

MK showed toxicity at concentrations higher than 125 µg/ml (Fig. S6). Using GraphPad Prism 9, the inhibitory IC_10_, IC_50_, and selectivity index (SI) were generated (Fig. S7). MK revealed a lower selectivity for both MCF-7 (1.3) and HeLa (1.1) cell lines in comparison to the Caco-2 cell line (1.5).

### Antidiabetic potential

The study found that the extracted MK inhibits α-glucosidase with concentration-dependent action (Fig. S8A)**.** The IC_50_ values for acarbose (standard) and MK were calculated as 22.77 and 57.15 µg/ml, respectively (Fig. S8B), which indicates that the extracted MK possesses less α-glucosidase inhibition than acarbose.

## Discussion

While microbes can live in a variety of ecological niches, the bacterial communities that are associated with them are typically different from the surrounding environment. They comprise taxa that differ significantly from free-living marine microorganisms, exhibit unique traits, and produce novel biologically active molecules that differ in structure and function [48–50].

According to a prior investigation by Metwally et al. [29], a bacterial strain known as *Kocuria* sp. RAM1 was reported as carotenoids promising producer. In addition to the carotenoids fraction, the yellow methanolic fraction was the focus of the current investigation. Liquid–liquid extraction is commonly used for MK extraction. The MK extraction from wet cells was reported and proved to give the highest recovery efficiency [31]. Upon methanol evaporation, it was observed that the extracted yellow fraction was slightly oily at room temperature after dryness, which agrees with the observation concerning pure MK compounds [51, 52]. Also, the UV spectrum with the characteristic absorbance has settled its identity as typical of MKs [32, 53]. Since it is significant to confirm the chemical contents, the use of an LC-QTOF-MS is one of the most successful procedures for mass estimation and compound determination [54]. Thus, the molecular weights of the six extracted compounds as MK analogs that vary in saturation were determined as MK-1, MK-3, MK-5 (H2), MK-7 (H6), MK-8 (H2) and MK-9. Q-TOF was performed three times to make sure there were no differences in the samples using the same extraction procedure, as it is well known that the purity profile of MK-7 can vary depending on methodologies and extraction procedures [55], As a result, we suspect that MKs are strongly pure, but we are seeking to validate this in a more advanced method.

According to Koehn et al. [4], similar MKs with one or more saturated isoprene units are present in certain bacteria, whether they are Gram-positive or Gram-negative. Chen et al. [30] reported that MK8 (H2)'s longer isoprenoid side chain and hydrogenation may enhance biological activity and stability in metabolic processes. In contrast with partial saturation, it is believed that the full saturation identified in archaea renders it more resistant to challenging conditions [56].

Previous investigations proved the determination of MK-7(H2), MK-8(H2) and MK-9(H2) in different *Kocuria* strains *such as K. aegyptia, K. rosea, K. kristinae, K. varians, K. palustris, K. rhizophila, K. polaris, K. carniphila, K. coralli, K. flava, K. turfanensis, K. salsicia, K. sediminis, K. tytonicola* [57–63]*.* Furthermore, MK derivatives with isoprenoid side chain lengths from MK-5 to MK-14 that were observed in different actinomycetes [32]. In thermophilic bacteria, it was reported that MK-11 is the major quinone in *Roseiflexus castenholzii* [64]**,** whereas MK-10 was the predominant quinone in *Chloroflexus* spp. and *Oscillochloris trichoides* [65, 66]. MK analogues, such as MK-8 and MK-9, were found mostly in six *Lactococcus lactis* strains, in addition to MK-5, MK-6, MK-7 and MK-10 [67]. Since there is currently no appropriate catalyst for selectively saturating a single isoprene unit while retaining the other double bonds unbroken, the synthesis of partially saturated MK analogues is still difficult [4]. So, in the current study it is interesting to extract different MKs derivatives that differ in saturation from *Kocuria* sp. RAM1.

Over the past two decades, using *B. subtilis, E. coli, and Lactococcus lactis,* numerous researchers have utilized culture optimization and metabolic engineering techniques to raise MK-7 production [68–71], but in this study it was important to discover another new bacterial strain with a variety of MK derivatives without gene modification. Thus, optimization is a necessary step to determine the key nutrients that can be identified from the screening study, and with CCI design implementation and RSM observation, the optimal fermentation media to improve MK production can be established.

According to our findings, the incubation temperature significantly impacted MK synthesis. Low MK was observed at high temperatures because of limited growth, which may be attributed to enzyme denaturation [67, 72]. Cultures grown at 180 rpm showed a significant increase in MK concentration owing to enhanced biomass accumulation because of increased air supply. In a study contrary to the present results, it was found that the highest obtained MKs produced by *L. lactis ssp. Cremoris* was at 60 rpm followed by 200 and 120 rpm. In similar, the highest concentration of MK by *L. lactis ssp. Cremoris* was in range of 30–33.5 °C, temperatures either above or below the mentioned range led to reduced MK synthesis [67]. In addition, peptone is a key nitrogen source that promoted *Kocuria* growth and MK production. This is due to its critical function in metabolism and protein building during the cellular respiration processes [28]. In a similar study, it is reported that peptone had a noticeable impact on MKs produced by *B. subtilis* when compared to tryptone, yeast extract, soy peptone [28]. *Bacillus subtilis natto* was investigated in many studies. A maximum MK-7 output of 62.32 mg/l was reported by Berenjian et al. (2011) after six days [73]. Chen et al. (2016) indicated that the ultimate output of MK-7 may attain 200–300 mg/l [15]. The production modules of MK-7 were developed by Cui et al. (2019) utilizing a dual-functional quorum-sensing mechanism in *B. subtilis* 168, achieving a final product of 360 mg/l MK-7 [12]. The biosynthesis of MK-7 increased to 410 mg/l after 6 days in shake-flask culture [14]. So, our MK output (394.69 mg/l) is considered close to the high previous values, without genetic modification.

For some of possible in vitro activities, our goal is to point out a novel mixture of MKs from marine *Kocuria* sp. RAM1 in this investigation. Considering the resistance acquired by pathogenic microorganisms, the development of new antimicrobial agents is a vital field of study. The extracted MK presented a minimal bactericidal concentration of 250 and 500 µg/ml. The MBC/MIC ratio can be used to determine whether MK is bacteriostatic or bactericidal. The effect is classified as bacteriostatic if the MBC/MIC ratio is greater than 4 and as bactericidal if it is less than 4 [74]. Hence, MKs have a bactericidal outcome. When MKs on Vero cells were tested, it was found that the extracted MKs are toxic to cells at concentrations greater than 125 µg/ml. Therefore, it is crucial to keep in mind that additional considerations, such as treatment duration, are necessary for in vivo testing at concentrations higher than 125 g/ml in the case of MK as an antibacterial.

Through studies related to COVID-19, it was concluded that many complications for intensive care patients were caused by the bacterial infection of *K. pneumoniae* (a multidrug-resistant bacteria), causing high death rates [75, 76]. So, it was interesting that the extracted MK can influence this type of bacteria. With the previously mentioned effect, the extracted MK also showed activity against *S. aureus, P. aeruginosa, E. faecalis,* and *E. coli.* This is important because of wound infections that require prompt treatment, particularly those caused by easily acquired antibiotic resistance mechanisms in hospitals [77–79], which led us to use the established MNTD of 125 µg/ml to assess the wound healing process.

MKs display antioxidant and prooxidant characteristics due to the redox features of the quinone ring. It was suggested that detection of different MK derivatives (MK-6, MK-8, MK-10, and MK-12) in previously unidentified components of buckwheat honey might play a role in its antioxidant and antibacterial activity [80]. According to Gao et al. [81], MK-4 slowed the progression of sepsis-associated acute lung injury (SI-ALI) by upregulating Sirt3 expression. This results from MK-4's antioxidant properties, which lower inflammatory factors and ROS levels. indicate that MK-4 is a potentially effective new treatment for sepsis and SI-ALI.

Conversely, MK may also have a role in prooxidant function. The conversion of quinone to semiquinone radical, followed by its interaction with molecular oxygen, can yield superoxide radical. This could subsequently boost prooxidant behavior and the generation of reactive oxygen species (ROS) through the Fenton mechanism. The prooxidant capabilities of MK and the synthesis of the cytotoxic ROS may be fundamental to MK's antibacterial efficacy. Schlievert et al. (2013) investigated MK analogs and found that their potent prooxidant activity, characterized by the formation of cytotoxic reactive oxygen species (ROS), damaged the integrity of the bacteria's cell envelope (plasma membranes) and disrupted bacterial respiration [82]. The current investigation demonstrated that MKs had antibacterial efficacy against both Gram-positive and Gram-negative bacteria. This contradicts the findings of Riffel et al. (2002), which indicated that naphthoquinone and menadione exhibited antibacterial action against Gram-positive bacteria, including *Staphylococcus aureus, Enterococcus faecium, and Bacillus subtilis*, but had no effect towards Gram-negative bacteria [83], and no antimicrobial activity of MK was proved at concentration from 0 to 200 µg/ml in 24-h cultures of *S. aureus* MN8 [82]. Hence, further investigation is necessary to determine MKs' pharmacological properties in vivo and their potential for antimicrobial therapy, given their efficacy against the isolates under test.

In our recent investigation, the extracted MK considerably shielded the human erythrocytes from the hypotonic solution. As a result of the many documented negative health issues related to NSAIDs (nonsteroidal anti-inflammatory drugs), it is preferable to recommend a safer alternative [84, 85]. In the current study, RBCs were used to test the inflammatory inhibition of the extracted MKs since their membrane behaves in a similar manner to the lysosomal membrane. So, the resultant RBC stabilization by MKs suggests that the lysosomal membrane will also be protected, giving rise to inflammation inhibition [86, 87]. Some synthesized MK and quinone carboxylic acids have been shown to have rat-liver lysosomal membrane stabilizing activity, which has been attributed to the structure side chain [88, 89]. Vitamin K has an essential role in modulating inflammation [90] and its consumption can reduce and suppress rheumatoid arthritis inflammations [91–93]. An earlier study proved that vitamin K2 has an important regulatory role in anti-inflammatory compound synthesis and proinflammatory cytokine expression related to lipid-overloaded human liver hepatocellular carcinoma cells [94].

Strong antioxidants are constantly sought after due to their benefits in protecting organisms from harmful oxidation, such as slowing or preventing cancer progression, cardiovascular disease, diabetes, atherosclerosis and metabolic disorders [95]. The MK radical-scavenging activity relies on their characteristic chemical structure due to the presence of the quinone head that provides electron transfer capacity. The oxidation and reduction of the quinone ring are reversible between its oxidized and reduced forms as a function of electron transportation. This step occurs between both the membrane-anchored proteins functioning to be electron acceptors with the respiratory electron transport system as donors. They also carry out a variety of vital biological processes, including growth regulation, antioxidant defense, and hormone signaling [90, 96, 97]. According to Li et al. [98], it is suggested the use of vitamin K1 and K2 can reduce the perinatal hypoxic-ischemic encephalopathy in rats.

The hunt for natural materials with strong healing qualities has been the focus of the last few decades. Wound healing is an extremely orchestrated action that involves four stages: hemostasis, inflammation, proliferation, and remodeling [99]. It is well known that there are ongoing efforts to find effective compounds for accelerating wound healing. Therefore, we evaluated the use of MK maximum non-toxic dose (125 µg/ml) in wound healing in two different types of cells (HSF and OEC), and the results were promising. This effect was also enhanced by the previously described antibacterial, antioxidant, and anti-inflammatory impacts, which are all advantageous with the inflammation stage. This may spur further in vivo investigations. Correspondingly, in animal models, MK as MK-7 plays a major role in wound healing by enhancing its pharmacological actions [100–102]. Others have found that MKs with varying chain lengths are more efficacious than vitamin K in the curative rat bioassay [103]. Pazyar et al*.* [104] explored the topical vitamin K administration in patients and observed the reduced healing duration in comparison to the control group. The wound healing potential is mainly attributed to the incorporation of vitamin K through several steps for the transformation of inactive glutamic acid residues into active γ-carboxyglutamic acid. This conversion activates the necessary coagulation factors that promote hemostasis and cell proliferation, thereby hastening the healing process [105–109]. Also, it was proven that vitamin K can inhibit inflammatory factor expression, causing inflammatory response inhibition [110–112]. In addition, the antioxidant potential of MKs accelerates the wound healing process [113].

Despite undeniable breakthroughs in cancer field studies, it remains the extensive cause of death. Recently, numerous natural products have been investigated. *Kocuria* sp. RAM1 MKs showed a higher selectivity against Caco-2 than that of MCF-7 and HeLa cell lines, all with SI > 1, indicating their anticancer activity, but extra investigations are required to determine both the detailed therapeutic mechanism and the clinical implications. In terms of structure, quinones' cytotoxicity can be explained by their ability to bind with cellular nucleophiles, which is facilitated by their capacity to induce oxidative stress through the redox cycle [114]. MKs have been found to have antiproliferative effects in colon cancer [115], acute myeloid and lymphoblastic leukemia [116, 117], hepatocellular carcinoma [118, 119], prostate cancer [120] and cervical carcinoma [121] by triggering both apoptotic and autophagic programmed cell death and also through activation mechanisms of apoptosis. It is unknown what mechanisms underpin MKs' anticancer activity in ALL. According to one study, MK acts as an oncoprotein inhibitor for cancer therapy by inhibiting MCM7, which causes various types of cancers with extreme malignancy when levels are exceeded. As a result, MKs may significantly reduce cancer-associated cell proliferation [122].

Globally, the number of people estimated to have diabetes in 2019 was 463 million, rising to 578 and 700 million by 2030 and 2045, respectively [123]. α-glucosidase inhibitors could control the exceeded level of plasma glucose resulting from carbohydrate diets in diabetic or prediabetic patients [124–126]. The present study showed that different concentrations of MK led to an increasing inhibition percentage, but lower than acarbose which is the most frequently used OHA (oral antihyperglycemic agent (OHA) [127]. This encourages us to use it in conjunction with acarbose and other therapies to investigate the combined effect in more in vivo research. Diabetes is also a risk factor for vitamin K deficiency, as it is proven by the fact that MK-7 supplementation in type II diabetes patients led to improvements of glycemic indices after 12 weeks [128, 129].

The supplement's vitamin K2 content varies from one brand to another. Adult men should consume 120 µg of vitamin K per day, while adult women should consume 90 µg [130]. A dosage of 50–120 µg of MK-7 supplement per day was suggested in additional investigation [131]. Heart-health advantages have also been demonstrated with MK-7 dosages ranging from 180 to 360 µg daily [132], and even at dosages of 15 mg three times daily, MK-4 supplementation does not cause hypercoagulation, hence it is a safe option[133].

Cao et al. (2020) noted that MK-1, MK-2, MK-3, and MK-5 have received insufficient attention [134], but in our study, MK-1 was present in excess, about 80% of the MK composition, which is a high percentage that may introduce the studied biological activities. The capacity of MK-1 to MK-10 to enhance vitamin K-dependent carboxylation was confirmed by Yen & Mack (1980) [135]; all MKs were found to be effective in promoting vitamin K-dependent carboxylation with the minimum concentration (0.1 µm). In a similar vein, Houser et al. (1978) proved that from MK-1 to MK-4 all had carboxylase activity, whereas menadione was inactive [136]. Despite analogous mechanisms of behavior, there are disparities in the biological function among different forms of vitamin K [137]. So, our extracted bacterial MKs need a more in-depth study in the future by focusing on the explanation of the relation among each of the kinds of vitamin K and their varying biological activity.

## Conclusion

A previously isolated marine *Kocuria* sp. RAM1 utilized in MK extraction. The in vitro cytotoxicity results do not necessarily correspond to in vivo toxicity. This may be ascribed to physiological, anatomical, pharmacodynamic, and pharmacokinetic factors in living organisms and cell cultures. Consequently, additional in vivo toxicity evaluations of MK fractions are necessary. In this study, MKs were extracted from a Red Sea bacterium, *Kocuria* sp. RAM1, and from our previous work, we have noticed that it is a promising strain. In this study, we extracted and optimized the production of MKs to 394.69 µg/ml. The chemical characterization of the total MKs confirmed the presence of six MKs analoges as MK-1 (the main component), MK-3, MK-5 (H2), MK-7 (H6), MK-8 (H2) and MK-9 with different saturation levels. We evaluated the impact of total MKs extract on many biological activities. MKs showed antibacterial activity against both Gram-positive and Gram-negative bacteria, which further need to be compared with other antibiotics, especially through in vivo studies. RBC membrane was stabilized by MKs, achieving the anti-inflammatory potential. The in vitro wound healing in both HSF and OEC, which is not previously reported, improved the wound closure rate when compared with the control samples. Also, MKs showed a cytotoxic potential against 3 cell lines (MCF-7, Caco-2 and HeLa). MKs showed less α-glucosidase inhibition than acarbose when evaluated as an antidiabetic agent. Also, our further studies will focus on the explanation of the fact that although different forms of vitamin K have similar mechanisms of action, their biological participation varies. Therefore, the extracted MKs are of therapeutic and medicinal interest, and we think these results may further encourage us to describe the mechanism for future in vivo studies.

## Supplementary Information

Supplementary materials 1. Table S1 Analysis of variance (ANOVA) of *Kocuria* sp. RAM1 MK using PBD. Table S2 Analysis of variance (ANOVA) for *Kocuria* sp. RAM1 MK using RSM. Fig. S1 PBD for MK optimization. Fig. S2 RSM factors interaction plots of MK. Fig. S3 Anti-inflammatory activity of MK indicated by hemolysis inhibition. Fig. S4 (A) Antioxidant activity of MK. (B) IC_50_ estimation. Fig. S5 MNTD estimation of MK; normal Vero cells cytotoxicity (A) and IC_50_ estimation (B). Fig. S6 Cytotoxicity of MK against three cancerous cell lines; MCF-7, Caco-2 and HeLa. Fig. S7 Cytotoxicity of MK against three cancerous cell lines (A: MCF-7, B: Caco-2 and C: HeLa) with IC_10_ and IC_50_ evaluation. Fig. S8 α-glucosidase inhibitory impact of MK vs. positive control; Acarbose at different concentrations (A) and IC_50_ evaluation (B)

## Data Availability

No datasets were generated or analysed during the current study.
