# Supplementary material for: Vitamin K (Menaquinone) from marine Kocuria sp. RAM1: optimization, characterization and potential in vitro biological activities
Source: Microb Cell Fact. 2025 Jun 7;24:132. doi: 10.1186/s12934-025-02751-8 (PMC12145650; doi:10.1186/s12934-025-02751-8)
Supplement: Supplementary file 1 — Supplementary materials 1. Table S1 Analysis of variance (ANOVA) of Kocuria sp. RAM1 MK using PBD. Table S2 Analysis of variance (ANOVA) for Kocuria sp. RAM1 MK using RSM. Fig. S1 PBD for MK optimization. Fig. S2 RSM factors interaction plots of MK. Fig. S3 Anti-inflammatory activity of MK indicated by hemolysis inhibition. Fig. S4 (A) Antioxidant activity of MK. (B) IC50 estimation. Fig. S5 MNTD estimation of MK; normal Vero cells cytotoxicity (A) and IC50 estimation (B). Fig. S6 Cytotoxicity of MK against three cancerous cell lines; MCF-7, Caco-2 and HeLa. Fig. S7 Cytotoxicity of MK against three cancerous cell lines (A: MCF-7, B: Caco-2 and C: HeLa) with IC10 and IC50 evaluation. Fig. S8 α-glucosidase inhibitory impact of MK vs. positive control; Acarbose at different concentrations (A) and IC50 evaluation (B) [file 12934_2025_2751_MOESM1_ESM.docx]

**Supporting Information**

**Vitamin K (Menaquinone) from marine *Kocuria* sp. RAM1: Optimization, characterization and potential *in vitro* activities**

**Rasha A. Metwally*^1^, Nermeen A. El-Sersy^1^, Amany El Sikaily^2^, Soraya A. Sabry^3^, Hanan A. Ghozlan^3^**

# *****[rashaabdou2012@hotmail.com](mailto:rashaabdou2012@hotmail.com)

**Table S1** Analysis of variance (ANOVA) of Kocuria sp. RAM1 MK using PBD.

| **Source** | **Sum of Squares** | **DF** | **Mean Square** | ***F*-value** | ***p*-value** |  |
| --- | --- | --- | --- | --- | --- | --- |
| **Model** | 1.226E+05 | 4 | 30648.43 | 19.10 | 0.0007 | **Significant** |
| **A-Peptone** | 9952.66 | 1 | 9952.66 | 6.20 | 0.0416 |  |
| **H-Temperature** | 65956.01 | 1 | 65956.01 | 41.11 | 0.0004 |  |
| **J-Agitation** | 23966.28 | 1 | 23966.28 | 14.94 | 0.0062 |  |
| **K-Inoculum Size** | 22718.76 | 1 | 22718.76 | 14.16 | 0.0070 |  |
| **Residual** | 11231.83 | 7 | 1604.55 |  |  |  |
| **Total** | 1.338E+05 | 11 |  |  |  |  |

**(R^2^ = 0.9161; Adj R² =0.8681; Pred R² = 0.7534)**

**Table S2** Analysis of variance (ANOVA) for *Kocuria* sp. RAM1 MK using RSM.

| **Source** | **Sum of Squares** | **df** | **Mean Square** | **F-value** | **p-value** |  |
| --- | --- | --- | --- | --- | --- | --- |
| Model | 1.934E+05 | 14 | 13816.16 | 15.67 | < 0.0001 | **Significant** |
| A-Peptone | 21408.43 | 1 | 21408.43 | 24.27 | 0.0002 |  |
| B-Temperature | 1.331E+05 | 1 | 1.331E+05 | 150.93 | < 0.0001 |  |
| C-Agitation | 16531.20 | 1 | 16531.20 | 18.74 | 0.0006 |  |
| D-Inoculum Size | 1.28 | 1 | 1.28 | 0.0015 | 0.9701 |  |
| AB | 4458.23 | 1 | 4458.23 | 5.06 | 0.0400 |  |
| AC | 1157.70 | 1 | 1157.70 | 1.31 | 0.2699 |  |
| AD | 8618.34 | 1 | 8618.34 | 9.77 | 0.0069 |  |
| BC | 97.22 | 1 | 97.22 | 0.1102 | 0.7445 |  |
| BD | 710.22 | 1 | 710.22 | 0.8053 | 0.3837 |  |
| CD | 2754.68 | 1 | 2754.68 | 3.12 | 0.0975 |  |
| **A²** | 2028.13 | 1 | 2028.13 | 2.30 | 0.1502 |  |
| **B²** | 52.60 | 1 | 52.60 | 0.0596 | 0.8104 |  |
| **C²** | 1391.86 | 1 | 1391.86 | 1.58 | 0.2282 |  |
| **D²** | 499.47 | 1 | 499.47 | 0.5663 | 0.4634 |  |
| **Residual** | 13228.74 | 15 | 881.92 |  |  |  |
| **Lack of Fit** | 7794.21 | 10 | 779.42 | 0.7171 | 0.6946 | not significant |
| **Pure Error** | 5434.53 | 5 | 1086.91 |  |  |  |
| **Cor Total** | 2.067E+05 | 29 |  |  |  |  |

**(R²** = 0.9360**; Adj R²** = 0.8762**; Pred R²** = 0.7449**; Adeq Precision** = 16.6225**)**

### **Optimization of MK production**

**
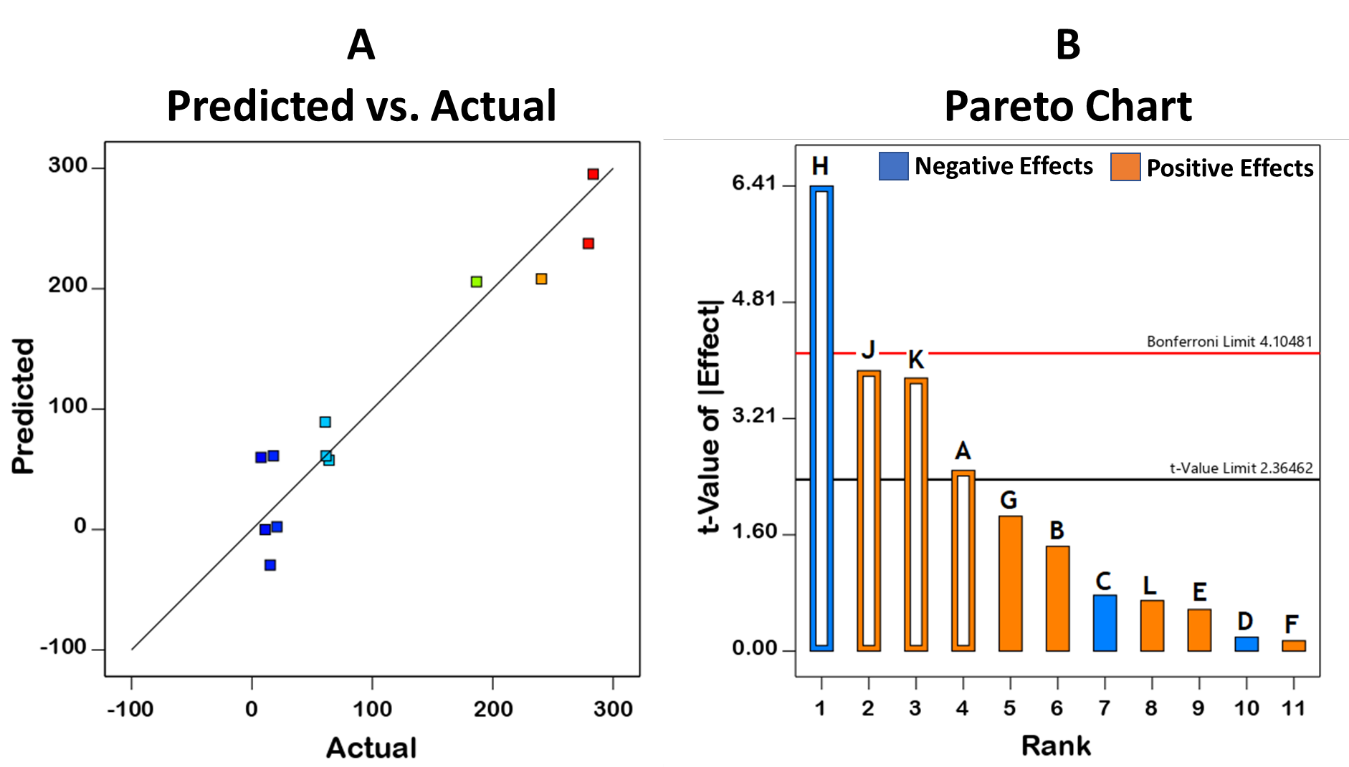
**

**Fig. S1** PBD for MK optimization. **(A)** The predicted value vs experimental value of MK. **(B)** MK output-affecting factors. The positive and negative responses are represented by the orange and blue bars. A, B, C, D, E and F (g/l) stand for peptone, yeast extract, beef extract, NaCl, glucose and MgSO_4_, respectively. G, H, J, K and L stand for pH, temperature (°C), agitation (rpm), inoculum size (%) and incubation period (h), respectively.

**
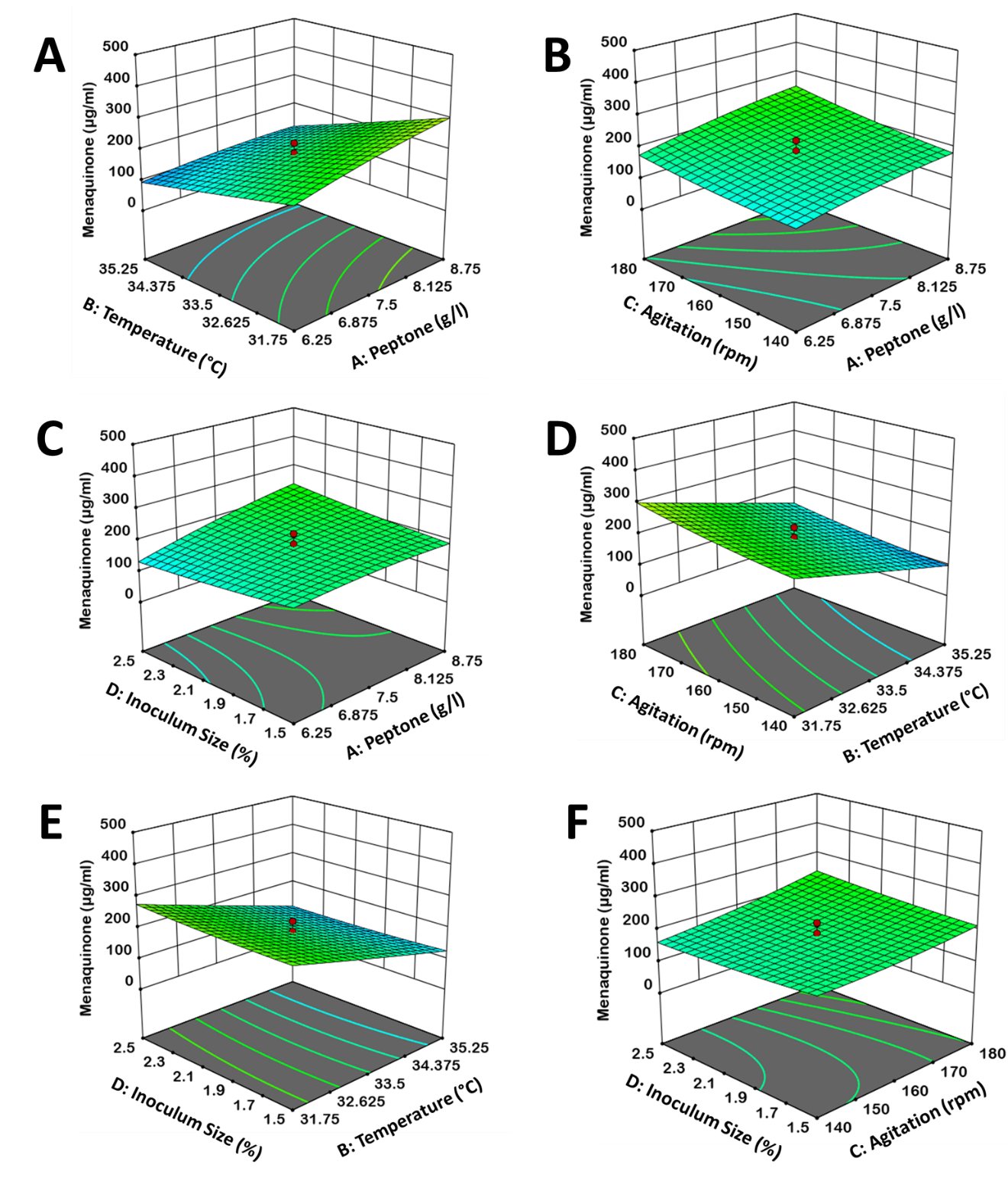
**

**Fig. S2** RSM factors interaction plots of MK.

### **Applications of MKs**

#### **Anti-inflammatory potential**

**Fig. S3** Anti-inflammatory activity of MK indicated by hemolysis inhibition. The mean ± SD_(n=3)_ is represented in the data.

#### **Antioxidant potential**

**
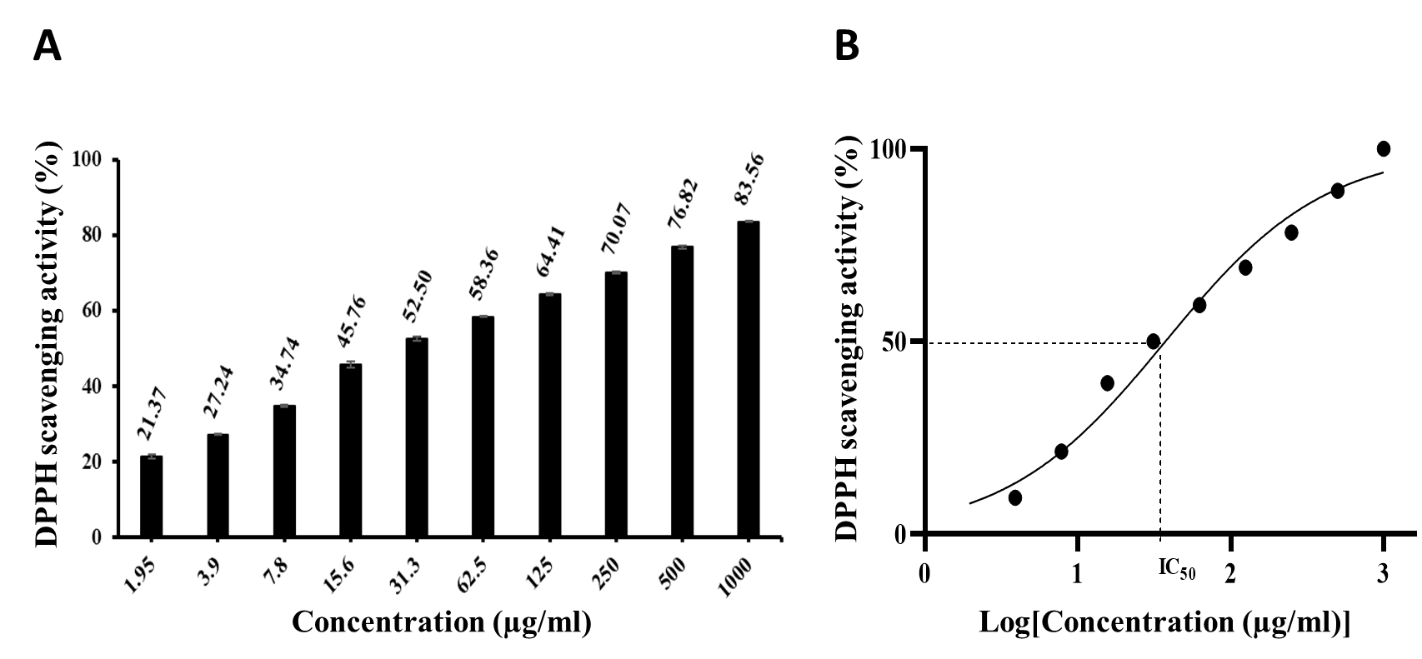
**

**Fig. S4** (A) Antioxidant activity of MK. (B) IC_50_ estimation. Mean ± SD _(n=3)_ is represented in the above data.

#### **The healing of wounds efficiency**


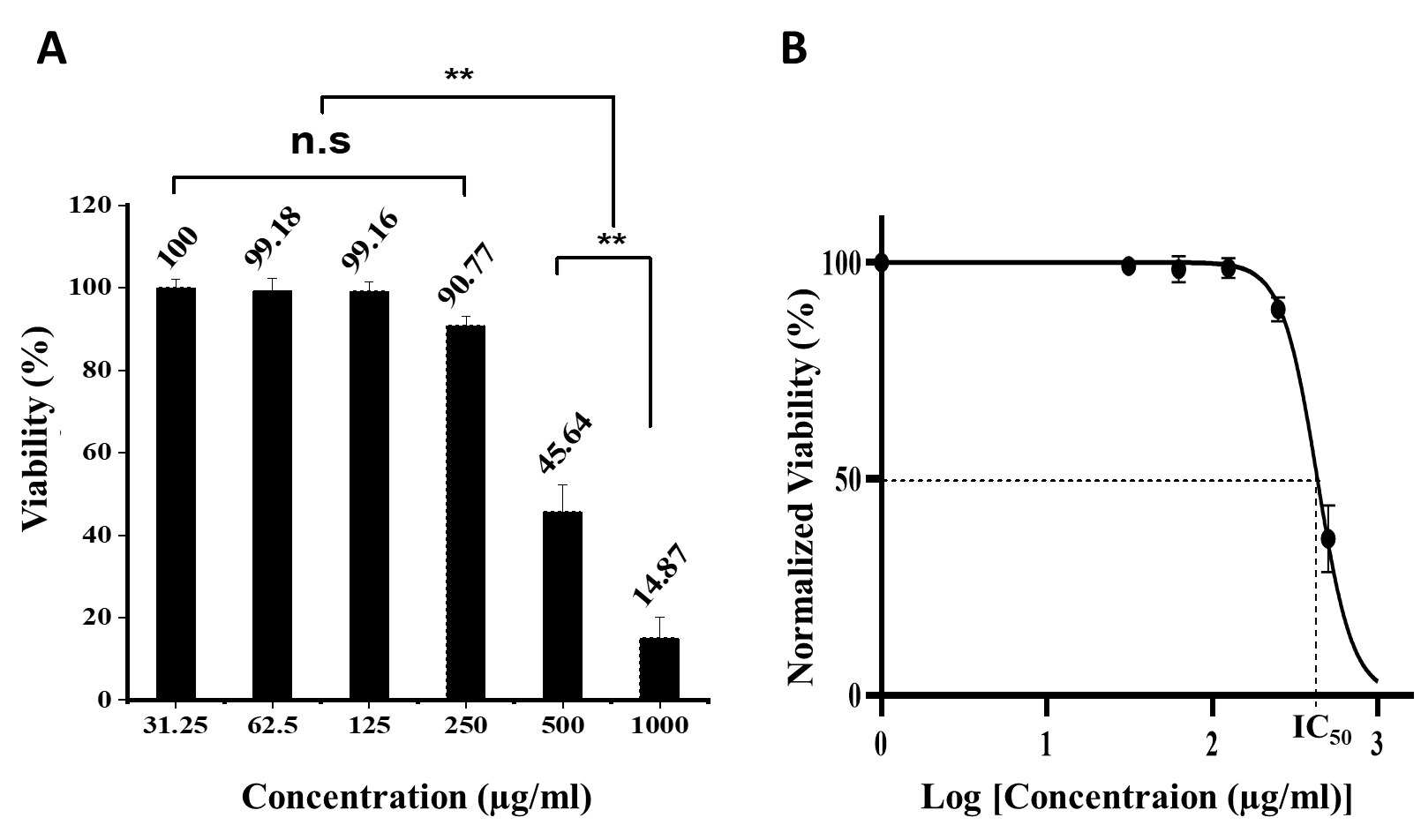


**Fig. S5** MNTD estimation of MK; normal Vero cells cytotoxicity (A) and IC_50_ estimation (B). Significance at *p* < 0.01 is symbolized by (**), while non-significance is symbolized by (n.s). Mean ± SD _(n=3)_ is represented in the data.

#### **Anticancer potential**


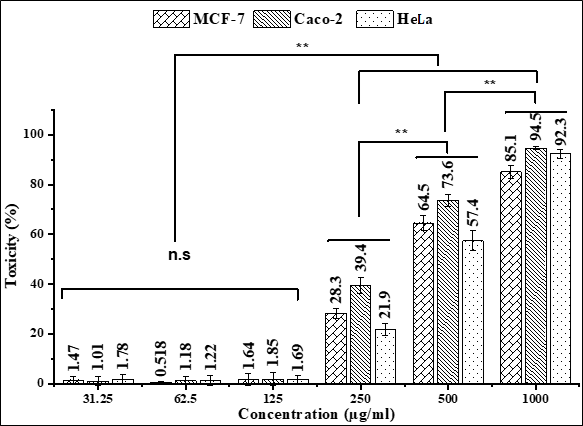


**Fig. S6** Cytotoxicity of MK against three cancerous cell lines; MCF-7, Caco-2 and HeLa. Significance at p < 0.01 is symbolized by (**), while non-significance is symbolized by (n.s). Mean ± SD _(n=3)_ is represented in the data.

**
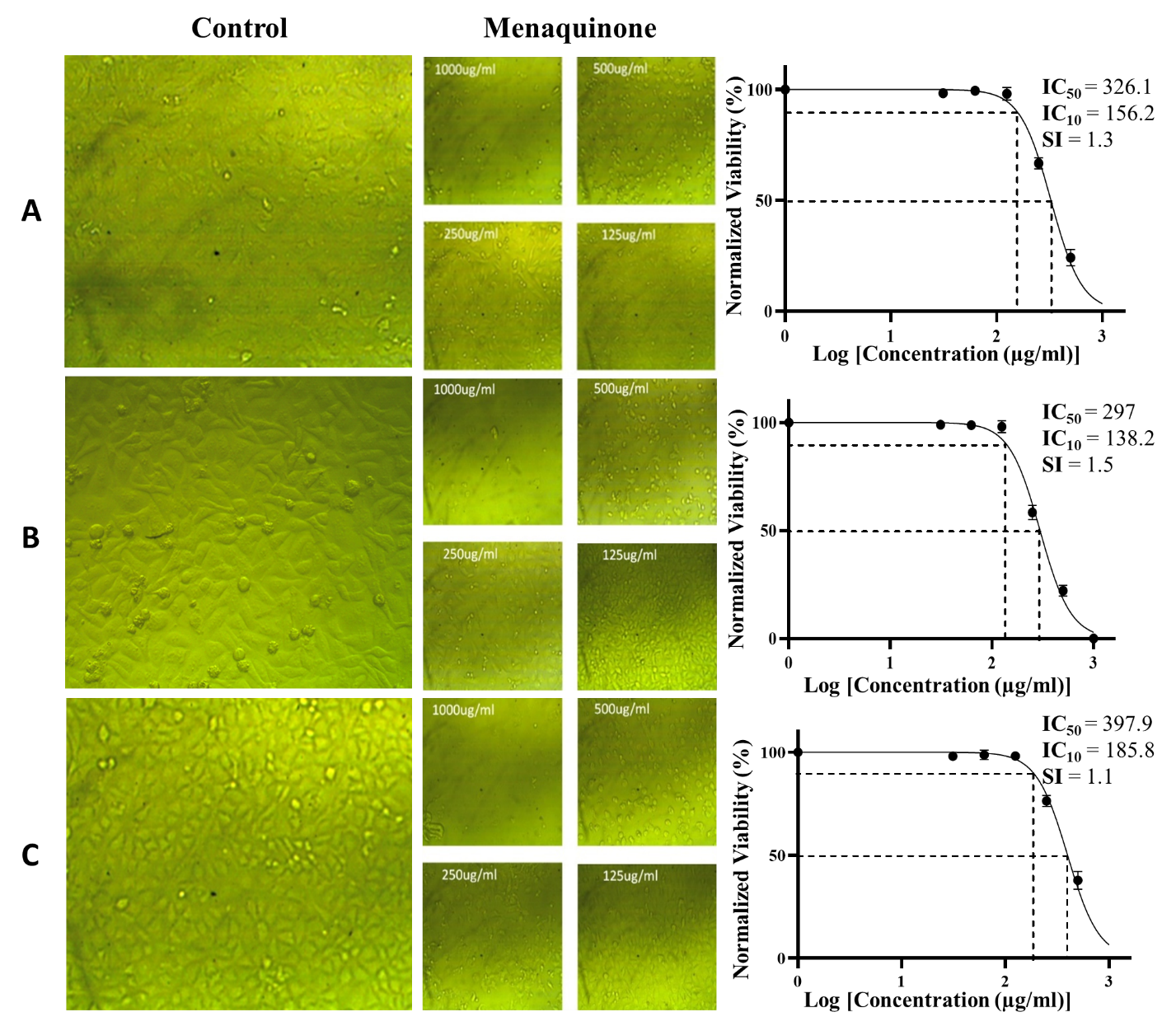
**

**Fig. S7** Cytotoxicity of MK against three cancerous cell lines (**A**: MCF-7, **B**: Caco-2 and **C**: HeLa) with IC_10_ and IC_50_ evaluation.

#### **Antidiabetic potential**

**
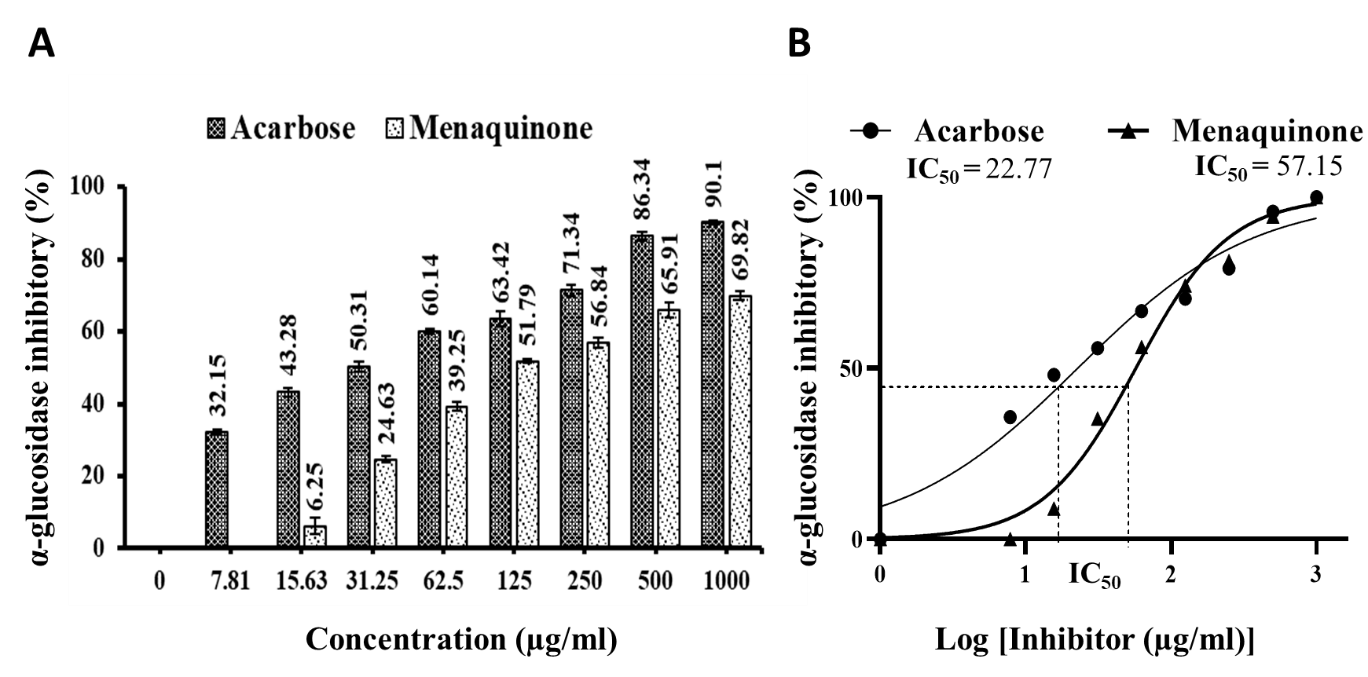
**

**Fig. S8** α-glucosidase inhibitory impact of MK vs. positive control; Acarbose at different concentrations (A) and IC_50_ evaluation (B). Mean ± SD _(n=3)_ is represented in the data.
